# Supplementary material for: Quantitative perturbation-based analysis of gene expression predicts enhancer activity in early Drosophila embryo
Source: eLife. 2016 May 6;5:e08445. doi: 10.7554/eLife.08445 (PMC4859806; doi:10.7554/eLife.08445)
Supplement: Supplementary file 1. — Restriction sites are capitals, with red capital letters indicating mutated binding site sequences. DOI: http://dx.doi.org/10.7554/eLife.08445.017 [file elife-08445-supp1.docx]

**Supplementary Table S1**

**Description and sequence information for constructs**

Restriction sites are capitalized, RED CAPITAL letters indicate mutated binding site sequences.

| **Construct No.** | **Construct Name and Description** | **Sequence** |
| --- | --- | --- |
| 1. | DMRW  (wt rhoNEE from *D. mel*) | 5’ - ACCGGTccttgggcaggatggaaaaatgggaaaacatgcggtgggaaaaacacacatcgcgaaacatttggcgcaacttgcg  gaagacaagtgcggctgcaacaaaaagtcgcgaaacgaaactctgggaagcggaaaaaggacaccttgctgtgcggcgggaa  gcgcaagtggcgggcggaatttcctgattcgcgatgccatgaggcactcgcatatgttgagcacatgttttgggggaaattcccgg  gcgacgggccaggaatcaacgtcctgtcctgcgtgggaaaagcccacgtcctacccacgcccactcggttacctGGCCGGCC – 3’ |
| 2. | DMRD1  (Dorsal1 site mutated) | 5’ – ACCGGTccttgggcaggatggaaaaatgggaaaacatgcggtTTTAAAAACACacatcgcgaaacatttggcgcaacttgcg  gaagacaagtgcggctgcaacaaaaagtcgcgaaacgaaactctgggaagcggaaaaaggacaccttgctgtgcggcgggaagcgcaagt  ggcgggcggaatttcctgattcgcgatgccatgaggcactcgcatatgttgagcacatgttttgggggaaattcccgggcgacgggccaggaatc  aacgtcctgtcctgcgtgggaaaagcccacgtcctacccacgcccactcggttacctGGCCGGCC – 3’ |
| 3. | DMRD2  (Dorsal2 site mutated) | 5’ – ACCGGTccttgggcaggatggaaaaatgggaaaacatgcggtgggaaaaacacacatcgcgaaacatttggcgcaacttgcggaagacaagtgcg  gctgcaacaaaaagtcgcgaaacgaaactctgggaagcggaaaaaggacaccttgctgtgcggcgggaagcgcaagtggcgggCGTCAGTTAATgat  tcgcgatgccatgaggcactcgcatatgttgagcacatgttttgggggaaattcccgggcgacgggccaggaatcaacgtcctgtcctgcgtgggaaaagcccac  gtcctacccacgcccactcggttacctGGCCGGCC – 3’ |
| 4. | DMRT1  (Twist1 site mutated) | 5’ – ACCGGTccttgggcaggatggaaaaatgggaaaacatgcggtgggaaaaacacacatcgcgaaacatttggcgcaacttgcggaagacaagtgcgg  ctgcaacaaaaagtcgcgaaacgaaactctgggaagcggaaaaaggacaccttgctgtgcggcgggaagcgcaagtggcgggcggaatttcctgattcgcgat  gccatgaggcactACGCGTTGTTgagcacatgttttgggggaaattcccgggcgacgggccaggaatcaacgtcctgtcctgcgtgggaaaagcccacgtc  ctacccacgcccactcggttacctGGCCGGCC – 3’ |
| 5. | DMRT2  (Twist2 site mutated) | 5’ – ACCGGTccttgggcaggatggaaaaatgggaaaacatgcggtgggaaaaacacacatcgcgaaacatttggcgcaacttgcggaagacaagtgcgg  ctgcaacaaaaagtcgcgaaacgaaactctgggaagcggaaaaaggacaccttgctgtgcggcgggaagcgcaagtggcgggcggaatttcctgattcgcgat  gccatgaggcactcgcatatgttgACGCGTTGTTttgggggaaattcccgggcgacgggccaggaatcaacgtcctgtcctgcgtgggaaaagcccacgtc  ctacccacgcccactcggttacctGGCCGGCC – 3’ |
| 6. | DMRD3  (Dorsal3 site mutated) | 5’ – ACCGGTccttgggcaggatggaaaaatgggaaaacatgcggtgggaaaaacacacatcgcgaaacatttggcgcaacttgcggaagacaagtgcggctg  caacaaaaagtcgcgaaacgaaactctgggaagcggaaaaaggacaccttgctgtgcggcgggaagcgcaagtggcgggcggaatttcctgattcgcgatgccat  gaggcactcgcatatgttgagcacatgttttggTCTAGATTATCgggcgacgggccaggaatcaacgtcctgtcctgcgtgggaaaagcccacgtcctaccca  cgcccactcggttacctGGCCGGCC – 3’ |
| 7. | DMRD4  (Dorsal4 site mutated) | 5’ – ACCGGTccttgggcaggatggaaaaatgggaaaacatgcggtgggaaaaacacacatcgcgaaacatttggcgcaacttgcggaagacaagtgcggctg  caacaaaaagtcgcgaaacgaaactctgggaagcggaaaaaggacaccttgctgtgcggcgggaagcgcaagtggcgggcggaatttcctgattcgcgatgccat  gaggcactcgcatatgttgagcacatgttttgggggaaattcccgggcgacgggccaggaatcaacgtcctgtcctgcgtAGGCCTGGTCAacgtcctacccac  gcccactcggttacctGGCCGGCC – 3’ |
| 8. | DMRD1D2  (Dorsal1 and Dorsal2 sites mutated) | 5’ – ACCGGTccttgggcaggatggaaaaatgggaaaacatgcggtTTTAAAAACACacatcgcgaaacatttggcgcaacttgcgg  aagacaagtgcggctgcaacaaaaagtcgcgaaacgaaactctgggaagcggaaaaaggacaccttgctgtgcggcgggaagcgcaagt  ggcgggCGTCAGTTAATgattcgcgatgccatgaggcactcgcatatgttgagcacatgttttgggggaaattcccgggcgacgggcca  ggaatcaacgtcctgtcctgcgtgggaaaagcccacgtcctacccacgcccactcggttacctGGCCGGCC - 3’ |
| 9. | DMRD1T1  (Dorsal1 and Twist1 sites mutated) | 5’ – ACCGGTccttgggcaggatggaaaaatgggaaaacatgcggtTTTAAAAACACacatcgcgaaacatttggcgcaacttgcgg  aagacaagtgcggctgcaacaaaaagtcgcgaaacgaaactctgggaagcggaaaaaggacaccttgctgtgcggcgggaagcgcaagtg  gcgggcggaatttcctgattcgcgatgccatgaggcactACGCGTTGTTgagcacatgttttgggggaaattcccgggcgacgggccagg  aatcaacgtcctgtcctgcgtgggaaaagcccacgtcctacccacgcccactcggttacctGGCCGGCC - 3’ |
| 10. | DMRD1T2  (Dorsal1 and Twist2 sites mutated) | 5’ – ACCGGTccttgggcaggatggaaaaatgggaaaacatgcggtTTTAAAAACACacatcgcgaaacatttggcgcaacttgcgg  aagacaagtgcggctgcaacaaaaagtcgcgaaacgaaactctgggaagcggaaaaaggacaccttgctgtgcggcgggaagcgcaagtg  gcgggcggaatttcctgattcgcgatgccatgaggcactcgcatatgttgACGCGTTGTTttgggggaaattcccgggcgacgggccagg  aatcaacgtcctgtcctgcgtgggaaaagcccacgtcctacccacgcccactcggttacctGGCCGGCC - 3’ |
| 11. | DMRD1D3  (Dorsal1 and Dorsal3 sites mutated) | 5’ – ACCGGTccttgggcaggatggaaaaatgggaaaacatgcggtTTTAAAAACACacatcgcgaaacatttggcgcaacttgcggaaga  caagtgcggctgcaacaaaaagtcgcgaaacgaaactctgggaagcggaaaaaggacaccttgctgtgcggcgggaagcgcaagtggcgggcgg  aatttcctgattcgcgatgccatgaggcactcgcatatgttgagcacatgttttggTCTAGATTATCgggcgacgggccaggaatcaacgtcctgt  cctgcgtgggaaaagcccacgtcctacccacgcccactcggttacctGGCCGGCC – 3’ |
| 12. | DMRD1D4  (Dorsal1 and Dorsal4 sites mutated) | 5’ – ACCGGTccttgggcaggatggaaaaatgggaaaacatgcggtTTTAAAAACACacatcgcgaaacatttggcgcaacttgcggaagacaag  tgcggctgcaacaaaaagtcgcgaaacgaaactctgggaagcggaaaaaggacaccttgctgtgcggcgggaagcgcaagtggcgggcggaatttcctg  attcgcgatgccatgaggcactcgcatatgttgagcacatgttttgggggaaattcccgggcgacgggccaggaatcaacgtcctgtcctgcgt  AGGCCTGGTCAacgtcctacccacgcccactcggttacctGGCCGGCC – 3’ |
| 13. | DMRD2T1  (Dorsal2 and Twist1 sites mutated) | 5’ –ACCGGTccttgggcaggatggaaaaatgggaaaacatgcggtgggaaaaacacacatcgcgaaacatttggcgcaacttgcggaagacaagtgcgg  ctgcaacaaaaagtcgcgaaacgaaactctgggaagcggaaaaaggacaccttgctgtgcggcgggaagcgcaagtggcgggCGTCAGTTAATgatt  cgcgatgccatgaggcactACGCGTTGTTgagcacatgttttgggggaaattcccgggcgacgggccaggaatcaacgtcctgtcctgcgtgggaaaag  cccacgtcctacccacgcccactcggttacctGGCCGGCC - 3’ |
| 14. | DMRD2T2  (Dorsal2 and Twist2 sites mutated) | 5’ – ACCGGTccttgggcaggatggaaaaatgggaaaacatgcggtgggaaaaacacacatcgcgaaacatttggcgcaacttgcggaagacaagtgcggctg  caacaaaaagtcgcgaaacgaaactctgggaagcggaaaaaggacaccttgctgtgcggcgggaagcgcaagtggcgggCGTCAGTTAATgattcgcgatg  ccatgaggcactcgcatatgttgACGCGTTGTTttgggggaaattcccgggcgacgggccaggaatcaacgtcctgtcctgcgtgggaaaagcccacgtcctac  ccacgcccactcggttacctGGCCGGCC - 3’ |
| 15. | DMRD2D3  (Dorsal2 and Dorsal3 sites mutated) | 5’ – ACCGGTccttgggcaggatggaaaaatgggaaaacatgcggtgggaaaaacacacatcgcgaaacatttggcgcaacttgcggaagacaagtgcgg  ctgcaacaaaaagtcgcgaaacgaaactctgggaagcggaaaaaggacaccttgctgtgcggcgggaagcgcaagtggcgggCGTCAGTTAATgattcg  cgatgccatgaggcactcgcatatgttgagcacatgttttggTCTAGATTATCgggcgacgggccaggaatcaacgtcctgtcctgcgtgggaaaagccca  cgtcctacccacgcccactcggttacctGGCCGGCC - 3’ |
| 16. | DMRD2D4  (Dorsal2 and Dorsal4 sites mutated) | 5’ – ACCGGTccttgggcaggatggaaaaatgggaaaacatgcggtgggaaaaacacacatcgcgaaacatttggcgcaacttgcggaagacaagtgcgg  ctgcaacaaaaagtcgcgaaacgaaactctgggaagcggaaaaaggacaccttgctgtgcggcgggaagcgcaagtggcgggCGTCAGTTAATgattc  gcgatgccatgaggcactcgcatatgttgagcacatgttttgggggaaattcccgggcgacgggccaggaatcaacgtcctgtcctgcgtAGGCCTGGTCA  acgtcctacccacgcccactcggttacctGGCCGGCC - 3’ |
| 17. | DMRT1T2  (Twist1 and Twist2 sites mutated) | 5’ –ACCGGTccttgggcaggatggaaaaatgggaaaacatgcggtgggaaaaacacacatcgcgaaacatttggcgcaacttgcggaagacaagtgcgg  ctgcaacaaaaagtcgcgaaacgaaactctgggaagcggaaaaaggacaccttgctgtgcggcgggaagcgcaagtggcgggcggaatttcctgattcgcga  tgccatgaggcactACGCGTTGTTgACGCGTTGTTttgggggaaattcccgggcgacgggccaggaatcaacgtcctgtcctgcgtgggaaaagccc  acgtcctacccacgcccactcggttacctGGCCGGCC - 3’ |
| 18. | DMRT1D3  (Twist1 and Dorsal3 sites mutated) | 5’ – ACCGGTccttgggcaggatggaaaaatgggaaaacatgcggtgggaaaaacacacatcgcgaaacatttggcgcaacttgcggaagacaagtgcgg  ctgcaacaaaaagtcgcgaaacgaaactctgggaagcggaaaaaggacaccttgctgtgcggcgggaagcgcaagtggcgggcggaatttcctgattcgcga  tgccatgaggcactACGCGTTGTTgagcacatgttttggTCTAGATTATCgggcgacgggccaggaatcaacgtcctgtcctgcgtgggaaaagccc  acgtcctacccacgcccactcggttacctGGCCGGCC – 3’ |
| 19. | DMRT1D4  (Twist1 and Dorsal4 sites mutated) | 5’ – ACCGGTccttgggcaggatggaaaaatgggaaaacatgcggtgggaaaaacacacatcgcgaaacatttggcgcaacttgcggaagacaagtgcgg  ctgcaacaaaaagtcgcgaaacgaaactctgggaagcggaaaaaggacaccttgctgtgcggcgggaagcgcaagtggcgggcggaatttcctgattcgcga  tgccatgaggcactACGCGTTGTTgagcacatgttttgggggaaattcccgggcgacgggccaggaatcaacgtcctgtcctgcgtAGGCCTGGTCA  acgtcctacccacgcccactcggttacctGGCCGGCC - 3’ |
| 20. | DMRT2D3  (Twist2 and Dorsal3 sites mutated) | 5’ – ACCGGTccttgggcaggatggaaaaatgggaaaacatgcggtgggaaaaacacacatcgcgaaacatttggcgcaacttgcggaagacaagtgcgg  ctgcaacaaaaagtcgcgaaacgaaactctgggaagcggaaaaaggacaccttgctgtgcggcgggaagcgcaagtggcgggcggaatttcctgattcgcga  tgccatgaggcactcgcatatgttgACGCGTTGTTttggTCTAGATTATCgggcgacgggccaggaatcaacgtcctgtcctgcgtgggaaaagccc  acgtcctacccacgcccactcggttacctGGCCGGCC - 3’ |
| 21. | DMRT2D4  (Twist2 and Dorsal4 sites mutated) | 5’ – ACCGGTccttgggcaggatggaaaaatgggaaaacatgcggtgggaaaaacacacatcgcgaaacatttggcgcaacttgcggaagacaagtgcgg  ctgcaacaaaaagtcgcgaaacgaaactctgggaagcggaaaaaggacaccttgctgtgcggcgggaagcgcaagtggcgggcggaatttcctgattcgcga  tgccatgaggcactcgcatatgttgACGCGTTGTTttgggggaaattcccgggcgacgggccaggaatcaacgtcctgtcctgcgtAGGCCTGGTCAa  cgtcctacccacgcccactcggttacctGGCCGGCC - 3’ |
| 22. | DMRD3D4  (Dorsal3 and Dorsal4 sites mutated) | 5’ –ACCGGTccttgggcaggatggaaaaatgggaaaacatgcggtgggaaaaacacacatcgcgaaacatttggcgcaacttgcggaagacaagtgcgg  ctgcaacaaaaagtcgcgaaacgaaactctgggaagcggaaaaaggacaccttgctgtgcggcgggaagcgcaagtggcgggcggaatttcctgattcgcga  tgccatgaggcactcgcatatgttgagcacatgttttggTCTAGATTATCgggcgacgggccaggaatcaacgtcctgtcctgcgtAGGCCTGGTCAa  cgtcctacccacgcccactcggttacctGGCCGGCC - 3’ |
| 23. | DMR4SM  (4 Snail sites mutated) | 5’ – ACCGGTccttgggcaggatggaaaaatgggaaaacatgcggtgggaaaaacacacatcgcgaaacatttggcgCAGAGCTCGGaagacaagtgcgg  ctgcaacaaaaagtcgcgaaacgaaactctgggaagcggaaaaaggaCAGGAGCTTGtgcggcgggaACGCCGGCGGcgggcggaatttcctgattcgc  gatgccatgaggcactcgcatatgttgaGCATATGTTTtgggggaaattcccgggcgacgggccaggaatcaacgtcctgtcctgcgtgggaaaagcccacgtc  ctacccacgcccactcggttacctGGCCGGCC - 3’ |
| 24. | DMRS1  (Snail2, Snail3 and Snail4 sites mutated) | 5’ – ACCGGTccttgggcaggatggaaaaatgggaaaacatgcggtgggaaaaacacacatcgcgaaacatttggcgcaacttgcggaagacaagtgcgg  ctgcaacaaaaagtcgcgaaacgaaactctgggaagcggaaaaaggaCAGGAGCTTGtgcggcgggaACGCCGGCGGcgggcggaatttcctgattcgc  gatgccatgaggcactcgcatatgttgaGCATATGTTTtgggggaaattcccgggcgacgggccaggaatcaacgtcctgtcctgcgtgggaaaagcccacgtc  ctacccacgcccactcggttacctGGCCGGCC - 3’ |
| 25. | DMRS2  (Snail1, Snail3 and Snail4 sites mutated) | 5’ – ACCGGTccttgggcaggatggaaaaatgggaaaacatgcggtgggaaaaacacacatcgcgaaacatttggcgCAGAGCTCGGaagacaagtgcgg  ctgcaacaaaaagtcgcgaaacgaaactctgggaagcggaaaaaggacaccttgctgtgcggcgggaACGCCGGCGGcgggcggaatttcctgattcgc  gatgccatgaggcactcgcatatgttgaGCATATGTTTtgggggaaattcccgggcgacgggccaggaatcaacgtcctgtcctgcgtgggaaaagcccacgtc  ctacccacgcccactcggttacctGGCCGGCC - 3’ |
| 26. | DMRS3  (Snail1, Snail2 and Snail4 sites mutated) | 5’ – ACCGGTccttgggcaggatggaaaaatgggaaaacatgcggtgggaaaaacacacatcgcgaaacatttggcgCAGAGCTCGGaagacaagtgcgg  ctgcaacaaaaagtcgcgaaacgaaactctgggaagcggaaaaaggaCAGGAGCTTGtgcggcgggaacgcaagtggcgggcggaatttcctgattcgc  gatgccatgaggcactcgcatatgttgaGCATATGTTTtgggggaaattcccgggcgacgggccaggaatcaacgtcctgtcctgcgtgggaaaagcccacgtc  ctacccacgcccactcggttacctGGCCGGCC - 3’ |
| 27. | DMRS4  (Snail1, Snail2 and Snail3 sites mutated) | 5’ –ACCGGTccttgggcaggatggaaaaatgggaaaacatgcggtgggaaaaacacacatcgcgaaacatttggcgCAGAGCTCGGaagacaagtgcgg  ctgcaacaaaaagtcgcgaaacgaaactctgggaagcggaaaaaggaCAGGAGCTTGtgcggcgggaACGCCGGCGGcgggcggaatttcctgattcg  cgatgccatgaggcactcgcatatgttgagcacatgttttgggggaaattcccgggcgacgggccaggaatcaacgtcctgtcctgcgtgggaaaagcccacg  tcctacccacgcccactcggttacctGGCCGGCC - 3’ |
| 28. | DMRS1S2  (Snail3 and Snail4 sites mutated) | 5’ – ACCGGTccttgggcaggatggaaaaatgggaaaacatgcggtgggaaaaacacacatcgcgaaacatttggcgcaacttgcggaagacaagtgcgg  ctgcaacaaaaagtcgcgaaacgaaactctgggaagcggaaaaaggacaccttgctgtgcggcgggaACGCCGGCGGcgggcggaatttcctgattcgc  gatgccatgaggcactcgcatatgttgaGCATATGTTTtgggggaaattcccgggcgacgggccaggaatcaacgtcctgtcctgcgtgggaaaagcccacgt  cctacccacgcccactcggttacctGGCCGGCC - 3’ |
| 29. | DMRS1S3  (Snail2 and Snail4 sites mutated) | 5’ – ACCGGTccttgggcaggatggaaaaatgggaaaacatgcggtgggaaaaacacacatcgcgaaacatttggcgcaacttgcggaagacaagtgcgg  ctgcaacaaaaagtcgcgaaacgaaactctgggaagcggaaaaaggaCAGGAGCTTGtgcggcgggaacgcaagtggcgggcggaatttcctgattcgc  gatgccatgaggcactcgcatatgttgaGCATATGTTTtgggggaaattcccgggcgacgggccaggaatcaacgtcctgtcctgcgtgggaaaagcccacgtc  ctacccacgcccactcggttacctGGCCGGCC - 3’ |
| 30. | DMRS1S4  (Snail2 and Snail3 sites mutated) | 5’ – ACCGGTccttgggcaggatggaaaaatgggaaaacatgcggtgggaaaaacacacatcgcgaaacatttggcgcaacttgcggaagacaagtgcgg  ctgcaacaaaaagtcgcgaaacgaaactctgggaagcggaaaaaggaCAGGAGCTTGtgcggcgggaACGCCGGCGGcgggcggaatttcctgattcgc  gatgccatgaggcactcgcatatgttgagcacatgttttgggggaaattcccgggcgacgggccaggaatcaacgtcctgtcctgcgtgggaaaagcccac  gtcctacccacgcccactcggttacctGGCCGGCC - 3’ |
| 31. | DMRS2S3  (Snail1 and Snail4 sites mutated) | 5’ –ACCGGTccttgggcaggatggaaaaatgggaaaacatgcggtgggaaaaacacacatcgcgaaacatttggcgCAGAGCTCGGaagacaagtgcgg  ctgcaacaaaaagtcgcgaaacgaaactctgggaagcggaaaaaggacaccttgctgtgcggcgggaacgcaagtggcgggcggaatttcctgattcg  cgatgccatgaggcactcgcatatgttgaGCATATGTTTtgggggaaattcccgggcgacgggccaggaatcaacgtcctgtcctgcgtgggaaaagcccacg  tcctacccacgcccactcggttacctGGCCGGCC - 3’ |
| 32. | DMRS2S4  (Snail1 and Snail3 sites mutated) | 5’ –ACCGGTccttgggcaggatggaaaaatgggaaaacatgcggtgggaaaaacacacatcgcgaaacatttggcgCAGAGCTCGGaagacaagtgcgg  ctgcaacaaaaagtcgcgaaacgaaactctgggaagcggaaaaaggacaccttgctgtgcggcgggaACGCCGGCGGcgggcggaatttcctgattcgc  gatgccatgaggcactcgcatatgttgagcacatgttttgggggaaattcccgggcgacgggccaggaatcaacgtcctgtcctgcgtgggaaaagcccacgt  cctacccacgcccactcggttacctGGCCGGCC - 3’ |
| 33. | DMRS3S4  (Snail1 and Snail2 sites mutated) | 5’ – ACCGGTccttgggcaggatggaaaaatgggaaaacatgcggtgggaaaaacacacatcgcgaaacatttggcgCAGAGCTCGGaagacaagtgcgg  ctgcaacaaaaagtcgcgaaacgaaactctgggaagcggaaaaaggaCAGGAGCTTGtgcggcgggaacgcaagtggcgggcggaatttcctgattcgc  gatgccatgaggcactcgcatatgttgagcacatgttttgggggaaattcccgggcgacgggccaggaatcaacgtcctgtcctgcgtgggaaaagcccacgtc  ctacccacgcccactcggttacctGGCCGGCC - 3’ |
| 34. | DMRB  (Both bHLH sites mutated) | 5’ – ACCGGTccttgggcaggatggaaaaatgggaaaacatgcggtgggaaaaacacacatcgcgaaaTGATTCgcgcaacttgcggaagaTAGCGAcgg  ctgcaacaaaaagtcgcgaaacgaaactctgggaagcggaaaaaggacaccttgctgtgcggcgggaagcgcaagtggcgggcggaatttcctgattcgcgatgcca  tgaggcactcgcatatgttgagcacatgttttgggggaaattcccgggcgacgggccaggaatcaacgtcctgtcctgcgtgggaaaagcccacgtcctacccacgccc  actcggttacctGGCCGGCC - 3’ |
| 35. | DMRD1B  (Both bHLH sites and Dorsal1 site mutated) | 5’ –ACCGGTccttgggcaggatggaaaaatgggaaaacatgcggtTTTAAAAACACacatcgcgaaaTGATTCgcgcaacttgcggaagaTAGCGAcgg  ctgcaacaaaaagtcgcgaaacgaaactctgggaagcggaaaaaggacaccttgctgtgcggcgggaagcgcaagtggcgggcggaatttcctgattcgcgatgccatg  aggcactcgcatatgttgagcacatgttttgggggaaattcccgggcgacgggccaggaatcaacgtcctgtcctgcgtgggaaaagcccacgtcctacccacgcccactcg  gttacctGGCCGGCC - 3’ |
| 36. | DMRBT2  (Both bHLH sites and Twist2 site mutated) | 5’ – ACCGGTccttgggcaggatggaaaaatgggaaaacatgcggtgggaaaaacacacatcgcgaaaTGATTCgcgcaacttgcggaagaTAGCGAcgg  ctgcaacaaaaagtcgcgaaacgaaactctgggaagcggaaaaaggacaccttgctgtgcggcgggaagcgcaagtggcgggcggaatttcctgattcgcgatgcca  tgaggcactcgcatatgttgACGCGTTGTTttgggggaaattcccgggcgacgggccaggaatcaacgtcctgtcctgcgtgggaaaagcccacgtcctacccacg  cccactcggttacctGGCCGGCC - 3’ |
| 37. | DMRBD3  (Both bHLH sites and Dorsal3 site mutated) | 5’ –ACCGGTccttgggcaggatggaaaaatgggaaaacatgcggtgggaaaaacacacatcgcgaaaTGATTCgcgcaacttgcggaagaTAGCGAcgg  ctgcaacaaaaagtcgcgaaacgaaactctgggaagcggaaaaaggacaccttgctgtgcggcgggaagcgcaagtggcgggcggaatttcctgattcgcgatgcc  atgaggcactcgcatatgttgagcacatgttttggTCTAGATTATCgggcgacgggccaggaatcaacgtcctgtcctgcgtgggaaaagcccacgtcctaccca  cgcccactcggttacctGGCCGGCC - 3’ |
| 38. | DMRBD4  (Both bHLH sites and Dorsal4 site mutated) | 5’ –ACCGGTccttgggcaggatggaaaaatgggaaaacatgcggtgggaaaaacacacatcgcgaaaTGATTCgcgcaacttgcggaagaTAGCGAcgg  ctgcaacaaaaagtcgcgaaacgaaactctgggaagcggaaaaaggacaccttgctgtgcggcgggaagcgcaagtggcgggcggaatttcctgattcgcgatgcc  atgaggcactcgcatatgttgagcacatgttttgggggaaattcccgggcgacgggccaggaatcaacgtcctgtcctgcgtAGGCCTGGTCAacgtcctacccac  gcccactcggttacctGGCCGGCC - 3’ |
| 39. | DMBRKS  (*D.mel* *brk* enhancer – 309 bp) | 5’ – ACCGGTgggaaatccaaaacacaacccgagcccgatccttcgctccttcgatttaagccaaagttagaggcacaggcacacatgtgtgtttggtttgaacgggaaag  ccccattttaaagctggccaaccaacggcaacacatgttcatgttaggaccgatacaggttgacattccctggaaggatgcacctctgggagattcccacaaccggcagcagg  tcatgtccaaccgatcgttgcgggagccacttgtcccgaaaaaatccaaagaaactatcaagtggcgtttagggaaactcaGGCCGGCC - 3’ |
| 40. | DMBRKL  (*D.mel* *brk* enhancer – 649 bp) | 5’ – ACCGGTaacaggtactacgatgatattggtcggaaaatacctgcgcatcctggtggtttatggtgcggccgtaaatgcaagccaagttctttacggcttctctggca  caaaccctaaatgtggattacgctaatattgccccccctaataaaaacggtcgttgtccagggccgaatattgcgtctgattggtttttcccacgattacaattagccggacgg  acacaaactgacctgagctgacccgcaaaaagacacggttgtccggcagtcggaactgaaggaaactaaaggaaactgagggcaggtcagcgctatggattgtgcactaa  gttgcttaatccgacgggaaatccaaaacacaacccgagcccgatccttcgctccttcgatttaagccaaagttagaggcacaggcacacatgtgtgtttggtttgaacggga  aagccccattttaaagctggccaaccaacggcaacacatgttcatgttaggaccgatacaggttgacattccctggaaggatgcacctctgggagattcccacaaccggcagc  aggtcatgtccaaccgatcgttgcgggagccacttgtcccgaaaaaatccaaagaaactatcaagtggcgtttagggaaactcaGGCCGGCC - 3’ |
| 41. | DMVNS  (*D.mel* *vn* enhancer – 341 bp) | 5’ – ACCGGTgggcatttcacttacctgcgtgggaaaatcgactaatctgcgaccgccccgaggagtcagtttttgtttttagagcggtaaaggacaggtaacgggccacat  gtctggccggaaattccccgttgacccctgaccccgtgtccttatgacgaattcgtcacttggcgtgagcacacctggatttcccaccgcttagccagcggaaattccaaaacac  ctccggcccactggccctcaaaattgttatatgctctgctacgatgaagcagaagcagaagcagcagtgttttattggcggaagcatccgccaaattgcaccc  aatctgcGGCCGGCC - 3’ |
| 42. | DMVNL  (*D.mel* *vn* enhancer – 869 bp) | 5’ – ACCGGTcaagttgagaaatttgcctttgatatcgaccacatgtgtgcacagcgaaaaaatattaggtggaaagttgaaaatattccgaattattcacaatatcatctg  caggattcttatttttaaaagcttccgatattacaaaaacactttcttggttgtaaatattttactgaatttgtgtaatttttctgtgtgcaaatatgcagccagttctggatcttccg  aatcaccctgccttcgcgttttgcaccccgtcgctgtggagccatattttctttttagctgacgatttagtccatttcccgctcataatcgcatgaagttgtttgcctccaccgaatg  gcttaatccgccagatcgatgcgcctgtgttgactcaataattccctaacaactctttttacgcattttattgaaagtgccgaagttagcgggcatttcacttacctgcgtgggaa  aatcgactaatctgcgaccgccccgaggagtcagtttttgtttttagagcggtaaaggacaggtaacgggccacatgtctggccggaaattccccgttgacccctgaccccgt  gtccttatgacgaattcgtcacttggcgtgagcacacctggatttcccaccgcttagccagcggaaattccaaaacacctccggcccactggccctcaaaattgttatatgctct  gctacgatgaagcagaagcagaagcagcagtgttttattggcggaagcatccgccaaattgcacccaatctgcagtttgaagtgctcaaaacccccaccgctcccctgtgaat  ttccgccggccggcaaggtgaccgtgtgctaaaacaaaatttttatatcgaaattgccgGGCCGGCC - 3’ |
| 43. | DMVNDS  (*D.mel* *vnd* enhancer – 324 bp) | 5’ – ACCGGTagaaattcccgtaggtgagagccagggaaaccccaatcgggaatgacatgtgtacgacagacatgggactcagatgccttcgagatactggcgtcaca  ctgtctggcaatgggatttccgctcaggaggacggggaatgcccgtgtagcctgtccatagcgtgggaaattcgcgagtcggggtcttcgggaaaactcgaaatgggaaaa  ccggaagcaagcaaacttgcgccaacatgtggcacgacctgtttcgacccgtaaagagtccctgctgacctgtgctgacctgcactga  cccgaccaggtagGGCCGGCC - 3’ |
| 44. | DMVNDL  (*D.mel* *vnd* enhancer – 907 bp) | 5’ – ACCGGTcaccctgcgagcctctgcctccatattagtgtttagatcccatatagctgatggacttgtccggctaatcctggtagctctttaaattaaccaagcggg  cacagcgcgcaagtacaggacacagggtataattccccgcctctttgatctggcaagtactcaaggtcctggcgaatggcggttgggaaattctggcttgttgttcgaccctgg  cttagaaattcccgtaggtgagagccagggaaaccccaatcgggaatgacatgtgtacgacagacatgggactcagatgccttcgagatactggcgtcacactgtctggcaat  gggatttccgctcaggaggacggggaatgcccgtgtagcctgtccatagcgtgggaaattcgcgagtcggggtcttcgggaaaactcgaaatgggaaaaccggaagcaagc  aaacttgcgccaacatgtggcacgacctgtttcgacccgtaaagagtccctgctgacctgtgctgacctgcactgacccgaccaggtagctgcgatccttacgaggcggatttg  cgtttaattgttgatggtattaggcaaatcaaaactcggggtctgaccgggactaggtgtcaataatccagcgatttgggtgcacttattcaaagttaattccgggggaaatgtg  cgcgttttcggttccgaagcatgcctgcaggatgcacaccccccacctccttatcttcttaacaacggcaagtgcaaaaatctgtgaaagtcagagcgctacaggtagtgcagg  tagtttcctttgcatatcccgaccaacagggacctccttttgttaaaccttccggccattcacacgattgacacaggatgtcgctgcaataagcatgaaaca  gggaaaaatcgGGCCGGCC - 3’ |
| 45. | DMVND1  (*D.mel* *vnd* enhancer – 362 bp) | 5’ – ACCGGTgaccctggcttagaaattcccgtaggtgagagccagggaaaccccaatcgggaatgacatgtgtacgacagacatgggactcagatgccttcgaga  tactggcgtcacactgtctggcaatgggatttccgctcaggaggacggggaatgcccgtgtagcctgtccatagcgtgggaaattcgcgagtcggggtcttcgggaa  aactcgaaatgggaaaaccggaagcaagcaaacttgcgccaacatgtggcacgacctgtttcgacccgtaaagagtccctgctgacctgtgctgacctgcactgac  ccgaccaggtagctgcgatccttacgaggcggatttgcgGGCCGGCC 3’ |
| 46. | DMSOGLS  (*D.mel* *sog* enhancer – 406 bp) | 5’ – ACCGGTgttgccaatgccattgcgcatacgccgtgtcgtctatatggctatatggctatatggctgtatggtgcggggaaatccccgtaatcgcaggtagaattc  cagccggtgccgaggcgggacctgctcgcacctctaatcccgccagggttttcgggacatgggatattcccgacggcacagcatagcactccgttttcttttttttttttta  ttattattgtgtccagttttaatccggaaagcgggaattcccttccgctcgctgcctgcactgcgctgcgcagacgcatcggcgtccgtaagccgcttaccaaaaagata  cgggtatacccaaatggatgcctgcccatgtatatagaccattgggtggtatggaccatggaccataaagcGGCCGGCC - 3’ |
| 47. | DMSOGCE  (*D.mel* *sog* enhancer – 564 bp) | 5’ – ACCGGTtgtttatggcagccaattgatgccgactgacctgtgtgtgtgtgtgtgtgtgtgtggaagctcaggatggacagattcccgggtttcagcggaacaggta  ggctggtcgatcggaaattcccaccatacacatgtggctataatgccaacggcatcgaggtgcgaaaacagatgcagcctcataaaaggggcgcagataaggtcgc  ggttgcgtgggaaaagcccatccgaccaggaccaggacgaagcagtgcggttggcgcatcattgccgccatatctgctattcctacctgcgtggccatggcgatatc  cttgtgcaaggataaggagcggggatcataaaacgctgtcgcttttgtttatgctgcttatttaaattggcttcttggcgggcgttgcaacctggtgctagtcccaatccc  aatcccaattccaatccgtatacccgtatatccaatgcattctacctgtcctgggaatttccgatttggccgcacccatatggccacggatgcgtgagagtgctctccgt  gcgattctagatcatcGGCCGGCC - 3’ |
| 48. | DMTDP  (*D.mel* *twi* enhancer – 1123 bp) | 5’ –ACCGGTaagctcctaagtccaggtagttttgggacagggcaaaaccctgttggtggtttttctaaggggaccatttcgagtcctgggttttgctattacctaagccgg  cgatcggcgatctgcgatcggagatcttcgatcgtggttttttccagcggaagttcgcgctctgcattaatcgggtatttttggtggccccggcaggcaaacagataattata  tccggaaatttgacttttcgctcgtatttttctggattttcggagctccgagccgcattcgcctgcgattttctcggtacgtgtgtgtgggaattcactaattaggcataatgaa  accttttcgtggagttcccctcggttagggttgtggatttgcacgctttacgatggttggcaactaactgatgattatttaatagcggaatgatttcgatgggcgagcgtcta  aacatttcggcttgtttcctgggaaattcctgcgatcccaaagtatatacaaatggaaaatcctcgcacagcaaagttgattgggtaaatatgcaatcagatagatataa  cttataatcgatttatatatacttacatctaaatgaatttgatacccgatttatgtggattttcgtgtttttgatcaggggagattcattgcgtcttatttttttttttacaaaaag  aatataacttatttccatatcatttcaagtttttaagttaaattcttgtctaaacctaaactaacgctgcaaacaacaacattcaatgaagtttactaaatgttcaattgggaa  ttgcaaaacaaaatcttttctaccagaatgcaattttcaggaaatgcttttatgtaataaacataatttatcattactctgaatgcactttttcaaaacttagaaactctgtcct  atgaattcccgtcgatccaaagatattctcaatcccctttttgaatcaacaagtaaaatatttcaaaaattgccgacaattcccctcgtattcccc  gtcccgcatcccaacacgcatacttcccaggcattttcccaaatcgagagaaaacccaaagaataacccaagagaaacagaaaaatccaga  gcgtcgagtcaaggctctcttcaGGCCGGCC – 3’ |
| 49. | DMTDEPE  (*D.mel* *twi* enhancer – 764 bp) | 5’ –ACCGGTaagctcctaagtccaggtagttttgggacagggcaaaaccctgttggtggtttttctaaggggaccatttcgagtcctgggttttgct  attacctaagccggcgatcggcgatctgcgatcggagatcttcgatcgtggttttttccagcggaagttcgcgctctgcattaatcgggtatttttggtg  gccccggcaggcaaacagataattatatccggaaatttgacttttcgctcgtatttttctggattttcggagctccgagccgcattcgcctgcgattttct  cggtacgtgtgtgtgggaattcactaattaggcataatgaaaccttttcgtggagttcccctcggttagggttgtggatttgcacgctcctaggttaatta  aattcaatgaagtttactaaatgttcaattgggaattgcaaaacaaaatcttttctaccagaatgcaattttcaggaaatgcttttatgtaataaacataa  tttatcattactctgaatgcactttttcaaaacttagaaactctgtcctatgaattcccgtcgatccaaagatattctcaatcccctttttgaatcaacaagt  aaaatatttcaaaaattgccgacaattcccctcgtattccccgtcccgcatcccaacacgcatacttcccaggcattttcccaaatcgagagaaaaccca  aagaataacccaagagaaacagaaaaatccagagcgtcgagtcaaggctctcttcaGGCCGGCC – 3’ |
| 50. | DMTPE  (*D.mel* *twi* enhancer – 318 bp) | 5’ –ACCGGTgggaattgcaaaacaaaatcttttctaccagaatgcaattttcaggaaatgcttttatgtaataaacataatttatcattactctgaat  gcactttttcaaaacttagaaactctgtcctatgaattcccgtcgatccaaagatattctcaatcccctttttgaatcaacaagtaaaatatttcaaaaatt  gccgacaattcccctcgtattccccgtcccgcatcccaacacgcatacttcccaggcattttcccaaatcgagagaaaacccaaagaataacccaaga  gaaacagaaaaatccGGCCGGCC – 3’ |
| 51. | DMSPE  (*D.mel* *sna* enhancer – 457 bp) | 5’ – TTAATTAAggaggctgacatgcagactttgtacccggaaaaacagacaagcccgcatagccaagtccgattttccgcgtcgtcaaaaaaaaaaa  accaggcgcggtggtaaaattggaaaaaatccatagcaaacccaaaagttttgataaacccaagaggtgacaaaaaattccagcgcggaattccaattcccc  gcgatcctgcaaccaatggcgtgagggaagatgggaaatgtcgagccaataacccagccgcatttccatttcgtatttccctgcaatggattttccttcgacgaa  tgtccggatgtggatcagcgaaggatttaaagtaatttcccatcttatccaattacgcagtcagcgaaactcaagcaaaacaagccacgacgagtggaaaaac  tggctgggggttttctatatgtcctcacatcctacctggtgggtcGGCCGGCC – 3’ |
| 52. | DMSDP  (*D.mel* *sna* enhancer – 867 bp) | 5’ –ACCGGTaattgacaagaacaacaacaatgtctatggaaaatcgaacttcatcccagcacctgcagaaatcccgagcgagtcggggaaaaagtatttaa  cccccgaaagggttttccccaaaataatgaagtaatgaatgaagcggaaaacactggccgccaatctacctaatactaatgagcgggccaacccgaccaggaa  tttttgcaagtcaggtacttcaacggatatatgggttcgacaagtgcggattttcccgcgacatcaatgaggacttggccgggttatccgcggtgctcatcgggcaa  ttccgcggccgaggacttcatcgtagtgatcattaggtagatatgtgcatggatgtgacatggcgatcattgcgcggaataacacacgtaataaccgagatatccg  ggatgacccaccaggtaggatgtgaggacatatagaaaacccccagccagtttttccactcgtcgtggcttgttttgcttgagtttcgctgactgcgtaattggataa  gatgggaaattactttaaatccttcgctgatccacatccggacattcgtcgaaggaaaatccattgcagggaaatacgaaatggaaatgcggctgggttattggct  cgacatttcccatcttccctcacgccattggttgcaggatcgcggggaattggaattccgcgctggaattttttgtcacctcttgggtttatcaaaacttttgggtttgct  atggattttttccaattttaccaccgcgcctggttttttttttttgacgacgcggaaaatcggacttggctatgcgggcttgtctgtttttccgggtacaaagtctgcatgt  cagcctccGGCCGGCC – 3’ |
| 53. | DMSPEDE  (*D.mel* *sna* enhancer – 805 bp) | 5’ – ACCGGTaattgacaagaacaacaacaatgtctatggaaaatcgaacttcatcccagcacctgcagaaatcccgagcgagtcggggaaaaagt  atttaacccccgaaagggttttccccaaaataatgaagtaatgaatgaagcggaaaacactggccgccaatctacctaatactaatgagcgg  gccaacccgaccaggaatttttgcaagtcaggtacttcaacggatatatgggttcgacaagtgcggattttcccgcgacatcaatgaggacttg  gccgggttatccgcggtgctcatcgggcaattccgcggccgaggacttcatcgtagtgatcattCCTAGGTTAATTAAgacccaccagg  taggatgtgaggacatatagaaaacccccagccagtttttccactcgtcgtggcttgttttgcttgagtttcgctgactgcgtaattggataagatg  ggaaattactttaaatccttcgctgatccacatccggacattcgtcgaaggaaaatccattgcagggaaatacgaaatggaaatgcggctgggt  tattggctcgacatttcccatcttccctcacgccattggttgcaggatcgcggggaattggaattccgcgctggaattttttgtcacctcttgggttta  tcaaaacttttgggtttgctatggattttttccaattttaccaccgcgcctggttttttttttttgacgacgcggaaaatcggacttggctatgcgggct  tgtctgtttttccgggtacaaagtctgcatgtcagcctccGGCCGGCC – 3’ |
| 54. | DERW  (Putative *D.erecta* *rho*NEE – 261 bp) | 5’ – ACCGGTgggaaaaacacacatcgcgaaacatttggcggaacttgcggaagacaagtgcggctgcaacaaaaaaaagtcgcgaaccaaaact  ctgggaagcggaaaaaggacaccttgctgtgcggcgggaagcgcaagtggcgggcggaatttcctgattcccggcccatgaggcactcgcata  tgttgagcacatgttgggggaaattcccgggcgacgggccaggaatcaacgtcctgcgtgggaaaagccGGCCGGCC – 3’ |
| 55. | DARW  (Putative *D.ananassae* *rho*NEE – 328 bp) | 5’ – ACCGGTgggaaaaacacacatcccgccgcacacacacacacatcgcgaaacatttggcggaacttggcggagagacaagtgcc  gcaaaacaaaaagtcgcgaaataaaactccgggaagtgaaaatgtgtcagagtgggaaaaggaggcaaggacacaggacatacagga  catacaggacaccttgctgtgcggcgtgtgggaaacaagttgcggaatttcctggccaggaggcaagatagccgtcgcatatgttgagcac  atgttggaggaaattcccgcacaagggccaggaatcaacgtccggcgtgggaaaagcccGGCCGGCC – 3’ |
| 56. | DMOJRW  (Putative *D.mojavensis* *rho*NEE – 455 bp) | 5’ – ACCGGTgggaaaaccacactcacacacgtatacacacacagatacaaggactcacatagtttgtgggaactttcggacaagtgcaatacaaaagt  cgaagtcgcgaaaacgcgttgagcaattcaaatgaaaatccgcaatgcaacggaaggagcaaggacatcgcacatcgcacatcgcagaacctgcagc  aatcttcctgtgcggaaattcctgaatcgcacatgtggcacgcacatgttgctgctgcggcagtgggaaaaacgagacgacaaggaattccccgagagca  gctcgccatgccacgcctacacgcccacacacccagcaaggcggcaattatgagtacctgtgactgcaacttgcgacttgcctcacctgaagtgtggaggc  caaaaggtgaccgggacgtgcctcccagatttttgagagaacgtgggaaaaaagGGCCGGCC – 3’ |
| 57. | DGRW  (Putative *D.grimshawi* *rho*NEE – 315 bp) | 5’ – ACCGGTgggaaaaacacacatggactcacaggatgagcaattttgttgacaagtgcaacaaaagtcgccgaaatcgcgaaatgcgcttcacaatttcag  atgaaaatccgcaatgcaacggaagggagcaaggacaccgacgtagacgatgaacctgtgttcctgtcgcagcaacagcaacaaacaagtgcggaatttcct  gactccggacacatgtggcacgcacatgtttggcagagcgaaaaagaaacacacatcaaaaaggaattccccaggtcgagagagggaagcccacgcccaca  ggtgaGGCCGGCC – 3’ |
| 58. | DVRW  (*D.virilis* *rho*NEE – 315 bp) | 5’ – ACCGGTacgcacgcacacggcgatagaaattaacacgtagtttagcggaactttgtggcaagtgcaacaaaagtcgaagtcgcggacgattcaaatgaaaa  tctgcaatgctgcggaaggagcaaggacaacccacctgtctatgagtgtgcgagtgtgcgagtgtgtgtgtgtgtgtgcgagtgtgtgtgcgtgcgtgtgtgtgcaaca  agtgcggaaattcctgaatcgcacatgtggcacgcacatgtcgagcgggaaaaaaccgctcgatgctcggtccaaggaattccccgagccaagggaaGGCCGGCC – 3’ |
| 59. | DMTDE  (*D.mel* *twi* enhancer – 333 bp) | 5’ – ACCGGTggcaaaaccctgttggtggtttttctaaggggaccatttcgagtcctgggttttgctattacctaagccggcgatcggcgatctgcgatcggagatctt  cgatcgtggttttttccagcggaagttcgcgctctgcattaatcgggtatttttggtggccccggcaggcaaacagataattatatccggaaatttgacttttcgctcgt  atttttctggattttcggagctccgagccgcattcgcctgcgattttctcggtacgtgtgtgtgggaattcactaattagg  cataatgaaaccttttcgtggagttccccGGCCGGCC – 3’ |
